# Colocalization of the (Pro)renin Receptor/Atp6ap2 with H^+^-ATPases in Mouse Kidney but Prorenin Does Not Acutely Regulate Intercalated Cell H^+^-ATPase Activity

**DOI:** 10.1371/journal.pone.0147831

**Published:** 2016-01-29

**Authors:** Arezoo Daryadel, Soline Bourgeois, Marta F. L. Figueiredo, Ana Gomes Moreira, Nicole B. Kampik, Lisa Oberli, Nilufar Mohebbi, Xifeng Lu, Marcel E. Meima, A. H. Jan Danser, Carsten A. Wagner

**Affiliations:** 1 Institute of Physiology, University of Zurich, Zurich, Switzerland; 2 Divison of Nephrology, University Hospital Zurich, Zurich, Switzerland; 3 Division of Vascular Medicine and Pharmacology, Department of Internal Medicine, Erasmus Medical Center, Rotterdam, The Netherlands; University of Bern, SWITZERLAND

## Abstract

The (Pro)renin receptor (P)RR/Atp6ap2 is a cell surface protein capable of binding and non-proteolytically activate prorenin. Additionally, (P)RR is associated with H^+^-ATPases and alternative functions in H^+^-ATPase regulation as well as in Wnt signalling have been reported. Kidneys express very high levels of H^+^-ATPases which are involved in multiple functions such as endocytosis, membrane protein recycling as well as urinary acidification, bicarbonate reabsorption, and salt absorption. Here, we wanted to localize the (P)RR/Atp6ap2 along the murine nephron, exmaine whether the (P)RR/Atp6ap2 is coregulated with other H^+^-ATPase subunits, and whether acute stimulation of the (P)RR/Atp6ap2 with prorenin regulates H^+^-ATPase activity in intercalated cells in freshly isolated collecting ducts. We localized (P)PR/Atp6ap2 along the murine nephron by qPCR and immunohistochemistry. (P)RR/Atp6ap2 mRNA was detected in all nephron segments with highest levels in the collecting system coinciding with H^+^-ATPases. Further experiments demonstrated expression at the brush border membrane of proximal tubules and in all types of intercalated cells colocalizing with H^+^-ATPases. In mice treated with NH_4_Cl, NaHCO_3_, KHCO_3_, NaCl, or the mineralocorticoid DOCA for 7 days, (P)RR/Atp6ap2 and H^+^-ATPase subunits were regulated but not co-regulated at protein and mRNA levels. Immunolocalization in kidneys from control, NH_4_Cl or NaHCO_3_ treated mice demonstrated always colocalization of PRR/Atp6ap2 with H^+^-ATPase subunits at the brush border membrane of proximal tubules, the apical pole of type A intercalated cells, and at basolateral and/or apical membranes of non-type A intercalated cells. Microperfusion of isolated cortical collecting ducts and luminal application of prorenin did not acutely stimulate H^+^-ATPase activity. However, incubation of isolated collecting ducts with prorenin non-significantly increased ERK1/2 phosphorylation. Our results suggest that the PRR/Atp6ap2 may form a complex with H^+^-ATPases in proximal tubule and intercalated cells but that prorenin has no acute effect on H^+^-ATPase activity in intercalated cells.

## Introduction

The (pro)renin receptor (P)RR is a protein spanning the membrane once and with a large extracellular domain. The extracellular domain can be cleaved to yield a soluble, shorter fragment of approximately 28 kDa [1,2,3]. The (P)RR was initially identified as a receptor for renin and prorenin, inducing non-proteolytical activation of prorenin and thus allowing local production of angiotensin I from angiotensinogen by both renin and prorenin. In addition, binding of prorenin and renin may activate an angiotensin-independent intracellular signaling cascade leading to enhanced ERK1/2 phosphorylation [4].

(P)RR is identical to ATP6AP2, a protein that associates and co-immunoprecipitates with vacuolar-type H^+^-ATPases (V-ATPases) [5]. H^+^-ATPases are membrane-associated multi-protein complexes mediating the transport of protons by hydrolyzing ATP [6,7]. In the kidney, H^+^-ATPases are localized at the plasma membrane of most epithelial cells lining the nephron and mediate proton extrusion into urine or blood [8]. Moreover, H^+^-ATPases are found in many intracellular organelles such as endosomes and lysosomes and play there a critical role in endocytosis, e.g. receptor-mediated endocytosis in the proximal tubule [7,9]. The activity of plasma membrane-associated H^+^-ATPases is regulated by various hormones and factors including angiotensin II, aldosterone, acidosis or alkalosis [7]. Some of these effects are mediated by intracellular signaling cascades involving cAMP/PKA, PKC, ERK1/2 or AMPK [10,11,12,13,14]. Activation of these signaling pathways can result in enhanced trafficking and localization of H^+^-ATPases at the plasma membrane associated with increased activity. Disruption of signaling or the actin cytoskeleton-dependent trafficking reduces plasma membrane H^+^-ATPase localization and stimulation [15,16,17,18,19,20,21].

In various model organisms such as *Drosophila* or *Xenopus laevis* larvae, the (P)RR/Atp6ap2 is critical for fundamental cellular processes such as endocytic retrieval of proteins and Wnt signaling [22,23,24]. Whether these functions of the (P)RR/Atp6ap2 are related to its possible role as accessory subunit of the H^+^-ATPase or due to other functions has not been fully elucidated. However, endocytosis as well as Wnt signaling (e.g. the recycling of Wnt receptors) are sensitive to the disruption of other *bona fide* H^+^-ATPase subunits and H^+^-ATPase inhibitors providing a strong argument for a role of the (P)RR/Atp6ap2 in H^+^-ATPase trafficking, regulation, or function [22,24]. However, limited information is available about the localization of the (P)RR/Atp6ap2 in kidney, an organ with very intense expression of H^+^-ATPases, and whether H^+^-ATPase activity itself can be affected by acute application of prorenin.

The main questions addressed in this manuscript are 1) the localization of (P)RR/Atp6ap2 protein along the murine nephron and its colocalization with plasma membrane associated H^+^-ATPases, 2) the coregulation of (P)RR/Atp6ap2 and two major H^+^-ATPase subunits on mRNA and protein level, and 3) to test whether acute application of prorenin could regulate native plasma membrane H^+^-ATPase in intercalated cells in freshly isolated murine collecting ducts.

## Materials and Methods

### Animals

Experiments were performed in 8–12 weeks old male C57BL/6 (body weight 25–30 g) mice. All animal experiments were conducted according to Swiss laws for the welfare of animals and were approved by local authorities (Swiss Veterinary Authority of the Kanton Zurich, permission no 03/2011). The animals had free access to food and tap water. Where indicated NaCl (0.28 M), NaHCO_3_ (0.28 M), KHCO_3_ (0.28 M), or NH_4_Cl (0.28 M) were added to the drinking water for 7 days. Animals receiving the aldosterone analogue desoxycorticosterone acetate (DOCA) received subcutaneous injections at day 1 and 4 (2 mg/mouse). These treatments have been shown to induce metabolic acidosis or alkalosis in rodents and induce regulation of major transport proteins expressed in intercalated cells [25]. Each group consisted of at least 5 animals and was compared to the time-, age- and gender-matched corresponding control groups. All diets except of DOCA were given in drinking water supplemented with 1% sucrose and maintained on a standard diet. The control group received only 1% sucrose in drinking water.

For some experiments, mice were used expressing eGFP under the control of the Atp6v1b1 (B1) H^+^-ATPase subunit promoter inducing high levels of eGFP expression in intercalated cells along the collecting duct system (B1-eGFP mice) [26]. B1-eGFP mice were kindly provided by Dr. Lance Miller and Dr. Raoul Nelson, University of Utah, Salt Lake City.

### Isolation of mouse nephron segments and mRNA extraction

Defined segments of mouse nephrons were isolated from the kidneys of untreated male C57BL/6 mice or B1-eGFP, 10–12 weeks old using hand-dissection under a stereo microscope illuminated with normal light or fluorescent light (Leica M165FC).

mRNA extraction of organs and hand-dissected isolated nephron segments with subsequent quantitative real time RT-PCR was performed as described previously [27]. Enrichment of the hand-dissected nephron segment preparation was ensured, by testing each sample for the most dominantly expressed segment specific mRNA transcripts (Podoplanin, NaPi-IIa, NKCC2, NCC, AQP2, and Pendrin).

### RNA extraction from kidney and semi-quantitative RT-qPCR analysis

To determine (P)RR/ATP6AP2, ATP6V1B1, ATP6V0A4, and HPRT relative mRNA abundance in dissected tissues, total RNA was extracted from dissected kidney cortex and medulla using an RNeasy kit (Qiagen, Basel, Switzerland). RNA was bound to columns and treated with DNase for 15 min at room temperature to reduce genomic DNA contamination. Quantity and purity of total eluted RNA was assessed by spectrometry. To generate complementary DNA (cDNA), total RNA was reverse transcribed (RT reaction) by Taqman Reverse Transcription Kit (Applied Biosystems, USA). The thermal cycle conditions used were 25°C (10 min), 48°C (30 min) and 95° (5 min). Primers and probes were designed using Primer Express (Applied Biosystems, USA) and purchased from Microsynth, Switzerland (S1 Table). The specificity of the primers was tested using adult mouse kidney cDNA by conventional PCR. Each pair of primers resulted only in a single band of the expected size (data not shown). Probes were labelled with the reporter dye FAM at the 5ʹ end and the quencher dye TAMRA at the 3ʹ end. RT-PCR reactions were performed using Taqman Universal PCR Master Mix (Applied Biosystems, USA) 17 μl reactions were prepared using 3 μl of cDNA-template. Reactions were run in 96-well Optical reaction plates and caps (Applied Biosystems, USA). Thermal cycles were set at 50°C (2 min) 95°C (10 min) and then 40 cycles at 95°C (15 sec) and 60°C (1 min). Each reaction was made in triplicates and the average taken. Samples without enzyme in the RT reaction were used as negative controls to exclude contamination with genomic DNA. Only results with less than 1 cycle difference were taken into consideration. Cross point threshold (C_t_ value) was taken as the earliest cycle number in the PCR amplification, when fluorescence rises significantly above the background fluorescence.

The expression of candidate genes was normalized to the reference gene, HPRT giving comparable results and analyzed by the delta delta C_t_ method.

### MDCK cells and cell culture

MDCK cells (C11 clone, kindly provided by Dr. H. Oberleithner, University of Münster, Germany) [28] were cultured at 37°C and 5% CO_2_ in DMEM (no. E15-810, GE Healthare, Glattbrugg, Switzerland) supplemented with 10% heat-inactivated fetal bovine serum (FBS, Sigma-Aldrich, Buchs, Switzerland), 2 mM L-glutamine, and 1% non-essential amino acids (no. M11-003, GE Healthcare). After cells had reached 80–90% confluency, they were starved for 24 hrs and thereafter treated with the AT_1_R blocker losartan (10 μM, Sigma-Aldrich, Buchs Switzerland) and the AT_2_R blocker PD123319 (10 μM, Sigma-Aldrich, Buchs, Switzerland) for 30 min at 37°C followed by human prorenin (1 and 20 nM, a kind gift of Dr. Walter Fischli, Actelion, Allschwil, Switzerland) and angiotensin II (10 nM, Sigma-Aldrich, Buchs Switzerland) stimulation for 10 min.

### Membrane preparation from kidney and western blot analysis

For total membrane preparations, kidneys were dissected into cortex and medulla. Samples were homogenized in an ice-cold K-HEPES buffer (200 mM mannitol, 80 mM HEPES, 41 mM KOH, pH 7.5) containing a protease inhibitor mix (Complete Mini, Roche Diagnostics, Germany) at a final concentration of 1 tablet in a volume of 10 ml solution. Samples were centrifuged at 2000 rpm for 20 min at 4°C. Subsequently, the supernatant was transferred to a new tube and centrifuged at 41’000 rpm for 1 h at 4°C. The resultant pellet was resuspended in K-HEPES buffer containing protease inhibitors.

MDCK cells were lysed with ice-cold Radio-Immunoprecipitation Assay (RIPA) buffer (150 mM NaCl, 50 mM Tris, pH 7.4, 1% NP-40, 0.5% Na-Deoxycholate, 2 mM Phenylmethylsulfonylfluoride) supplemented with a protease inhibitor mix (Complete Mini, Roche Diagnostics, Germany, at a final concentration of 1 tablet in a volume of 10 ml solution) and incubated for 30 min on ice. Cellular debris was pelleted by centrifugation at 2500 g for 10 min at 4°C.

After measurement of the total protein concentration (Bio-Rad D_c_ protein Assay; Bio-Rad, Hercules, CA, USA), 10 μg of crude membrane proteins from cortex or medulla or 20 μg of MDCK extracts were solubilised in Laemmli buffer, and SDS-PAGE was performed on 10% polyacrylamide gels.

For immunoblotting, proteins were transferred electrophoretically to polyvinylidene difluoride membranes (Immobilon-P; Millipore, Bedford, MA, USA). After blocking with 5% milk powder in Tris-buffered saline/0.1% Tween-20 for 60 min; the blots were incubated with the respective primary antibodies: goat anti mouse (P)RR 1:1000, Novus Biologicals, USA (NB100-1318), rabbit anti mouse ATP6V1B1 1:5000 [29], rabbit anti-human ATP6V0A4 1:5000 [30], rabbit anti-pERK1/2 1:1000 (Cell Signaling, 9101, Danvers, MA, USA), rabbit anti-total ERK1/2 1:1000 (Cell Signaling, 9102, Danvers, MA, USA, and mouse monoclonal anti-β-actin antibody (42 kDa; Sigma, St. Louis, MO, USA) 1:5000, diluted in 1% milk/TBS-T) either for 2 h at room temperature or overnight at 4°C. After washing, the membranes were incubated for 1 h at room temperature with the secondary antibodies: donkey anti-goat, goat anti-rabbit and goat anti-mouse IgG-conjugated with alkaline phosphatase 1:5000 (Promega, WI, USA) and sheep anti-mouse IgG-conjugated with horseradish peroxidase (Amersham Life Sciences, 1:10000). Antibody binding was detected with enhanced chemiluminescence ECL kit (Amersham Pharmacia Biotech) or the CDP-Star Western chemiluminescence Kit (Roche Diagnostics, Mannheim, Germany) using the DIANA III-chemiluminescence detection system (Raytest; Straubenhardt, Germany). All images were analyzed using appropriate software (Advanced Image Data Analyzer, Raytest, Straubenhardt, Germany) to calculate the protein of interest/β-actin ratio.

### Immunohistochemistry

Mice were anesthetized with Ketamine/Xylazine and perfused through the left ventricle with phosphate-buffered saline (PBS) followed by paraformaldehyde-lysine-periodate (PLP) fixative [31]. Kidneys were removed and fixed overnight at 4°C by immersion in PLP. Kidneys were washed 3 times with PBS and 5 μm cryosections were cut after cryoprotection with 2.3 M sucrose in PBS for at least 12 h. Immunostaining was carried out as described previously [32,33,34]. Briefly, sections were incubated with 10 mM TRIS (Trizma Base, Sigma, pH 10 at 100°C for 20 min in a microwave, washed 3 times with PBS and incubated with 5% (v/v) donkey serum in PBS for 15 min prior to the primary antibody. The primary antibodies (goat anti-(P)RR (Novus Biologicals, USA) 1:100), rabbit anti-ATP6V0A4 (a4) serum 1:1000 [29,30], rabbit polyclonal anti ATP6V1B1 (B1) 1:150 [29], guinea-pig anti-pendrin 1:1000 [35], guinea-pig anti-AE1 1:500 [36], and rabbit-anti-AQP2 (kindly provided by J. Loffing, Zurich)[37] 1:1000 were diluted in PBS and applied either for 75 min at room temperature or overnight at 4°C. Sections were then washed twice for 5 min with high NaCl PBS (PBS + 18 g NaCl/l), once with PBS, and incubated with dilutions of the secondary antibodies (donkey anti-rabbit 586 (1:1000), donkey anti-goat 488 (1:1000), donkey anti-guinea-pig Dylight 649 (Jackson ImmunoResearch Lab Inc) (1:1000) mixed with DAPI (Molecular Probes, Oregon, USA) 1:1000 for 1 h at room temperature. Sections were again washed twice with high NaCl PBS and once with PBS before mounting with glycergel mounting medium (Dako, USA). Sections were viewed with a Leica DM5500B epifluorescence microscope and for images comparing localization and intensity of stainings pictures were taken on the same day and with identical settings for gain, intensity, and fluorescence filters. Images were processed (overlays) using Adobe Photoshop.

### *In vitro* microperfusion experiments

Mice were anesthetized with Xylazin/Ketamin i.p., both kidneys were cooled in situ with control bath solution containing in mM (138 NaCl, 1.5 CaCl_2_ 1.2 MgSO_4_, 2 K_2_HPO_4_, 10 HEPES, 5.5 glucose, 5 alanine, pH 7.4) for 1 min and then removed and cut into thin coronal slices for tubule dissection. Cortical collecting ducts (CCDs) were dissected under a stereo microscope from the cortex at 10°C in the control solution.

### Intracellular pH measurement

The isolated cortical collecting ducts were transferred into the bath chamber on the stage of an inverted microscope (IX81, Olympus, Japan) in the control solution and then mounted on concentric pipettes and perfused *ex vivo* with Na^+^-free, ammonium-free solution where N-methyl-D-Glutamine^+^ (NMDG^+^) replaced Na^+^. The average tubule length exposed to bath fluid was limited to 300–350 μm in order to prevent motion of the tubule. CCDs were loaded with 5 μM of the fluorescent probe BCECF-AM (2’,7’-bis(2-carboxyl)-5-(and-6)-carboxyfluorescein acetoxymethyl ester, Invitrogen, Switzerland) for ~20 min at 37°C in the control bath solution. The loading solution was then washed out by initiation of bath flow and the tubule was equilibrated with dye-free control bath solution for 5 min. Luminal incubation with prorenin (20 pM and 1 nM) was initiated during the BCECF loading and prorenin was presented throughout the incubation period and experiment (approx.40 min). Bath solution was delivered at a rate of 20 ml/min and warmed to 37°C by water jacket immediately upstream to the chamber. After temperature equilibration in control solution, tubules were first transiently acidified by peritubular Na^+^ removal (Na-free, ammonium-free solution) (10 min duration), where sodium was replaced by NMDG^+^ to avoid exit of NH_4_^+^ by basolateral Na^+^-coupled transport. This maneuver was done in the luminal absence of Na^+^. During the fluorescence recording, perfusion solution was delivered to the perfusion pipette via a chamber under an inert gas (N_2_) pressure (around 1 bar) connected through a manual 6-way valve. With this system, opening of the valve instantaneously activates flow of solutions. The majority of the fluid delivery to the pipette exits the rear of the pipette system through a drain port at 4 ml/min. This method results in a smooth and complete exchange of the luminal or the peritubular solution in less than 3 to 4 s [38].

After the fluorescence signal stabilization, luminal fluid was instantly (at the rate of 4 ml/min in the draining) replaced by a Na^+^-free solution containing 20 mM NH_4_Cl (and 118 mM NMDG-Cl) that elicited a rapid intracellular alkalinization, followed by a sharp acidification. The rate of intracellular alkalinization has been associated with the entry of NH_3_ whereas the subsequent phase of intracellular acidification in the continuous presence of extracellular NH_4_Cl reflects mostly NH_4_^+^ entry [39]. Intracellular dye was excited alternatively every 2 seconds at 434 and 494 nm with a MT fluorescence light source (150W Xenon/Mercury mixed gas burner) including a light guide and coupling to a disk scan Unit (Olympus, Japan). Emitted light was collected through a dichroïc mirror, passed through a 530 nm filter and focused onto a EM-CCD camera (Hamamatsu, Japan) connected to a computer. The measured light intensities were digitized with the Cell^M^&Cell^R^ Imaging hardware system (Olympus, Japan) for further analysis. Intracellular dye was calibrated at the end of each experiment using the high [K^+^]-nigericin technique. Tubules were perfused and bathed with a HEPES-buffered, 95-mM K^+^-solution containing 10 μM of the K^+^/ H^+-^exchanger nigericin. Four different calibration solutions, titrated to pH 6.3, 6.9, 7.5, or 7.8 were used.

### Statistical analysis

Data are provided as means ± SEM; *n* represents the number of independent experiments. All data were tested for significance using Student's unpaired two-tailed t-test, or ANOVA, where applicable. The level of statistical significance was set at * *p <* 0.05, ** *p <* 0.01 and *** *p <* 0.001.

## Results

### Localization of the (pro)renin receptor/Atp6ap2 in mouse kidney

**Fig 1:**
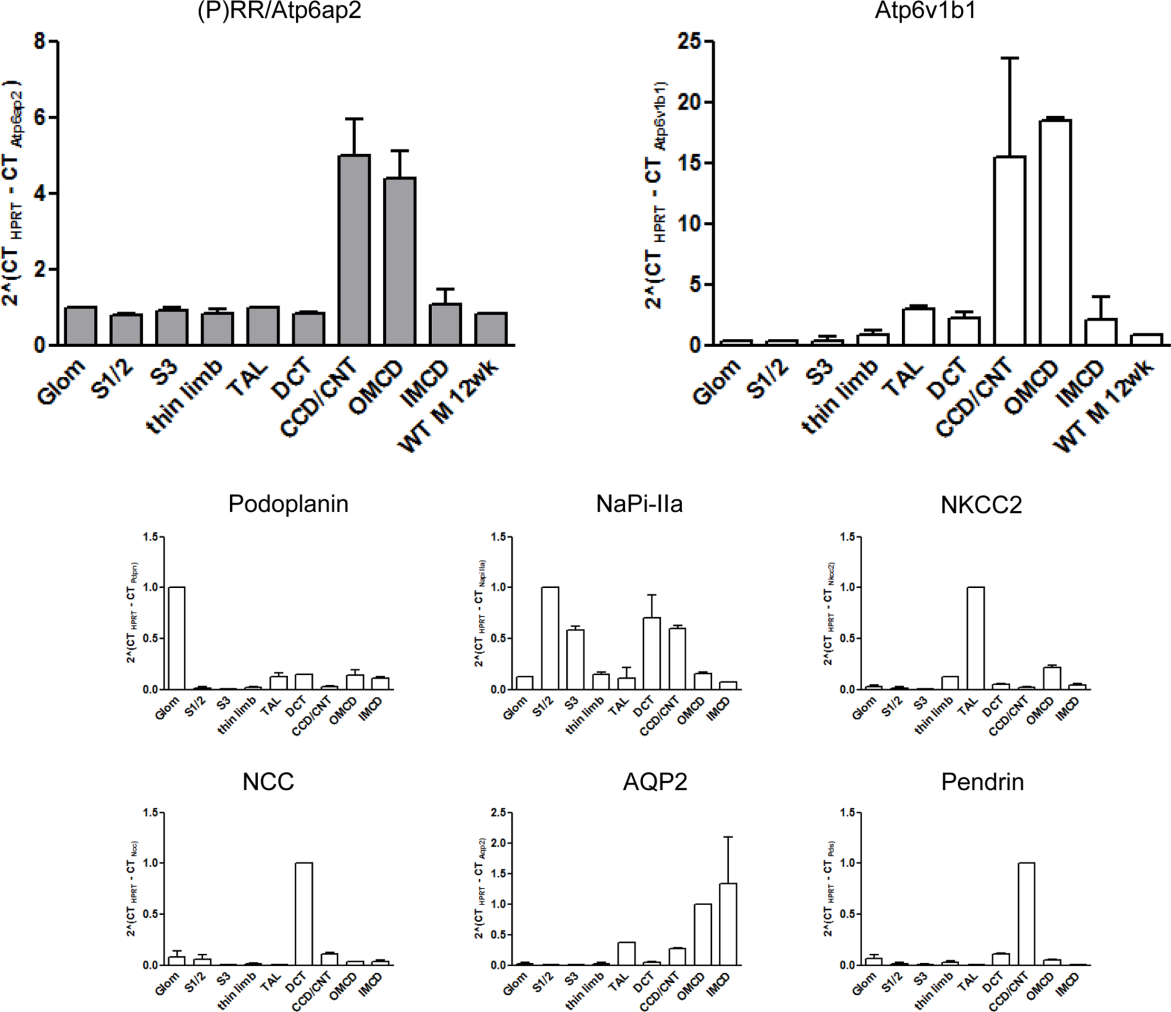
Expression of (P)RR/Atp6ap2 mRNA along the mouse nephron. Nephron segments were dissected from mouse kidney and relative mRNA abundance of the (P)RR and the B1 (Atp6v1b1) subunit of the vacuolar H^+^-ATPase assessed. Segment-specific enrichment of nephron fragments was tested by qPCR for various transcripts: Podoplanin for the glomerulum, NaPi-.IIa for the S1 /S2, S3 segments of the proximal tubule and DCT, NKCC2 for the TAL, NCC for the DCT, AQP2 for the collecting duct system, and pendrin for the CNT/CCD. Data are mean ± SEM (n = 4 mice/segments). Glom glomerulus, S1/S2 convoluted part of the proximal tubule, S3 straight part of the proximal tubule, thin limb thin descending and ascending limb of the loop of Henle, TAL thick ascending limb of the loop of Henle, DCT distal convoluted tubule, CNT/CCD connecting tubule/cortical collecting duct, OMCD outer medullary collecting duct, IMCD inner medullary collecting duct, WT M 12 wk C57BL/6 male mouse 12 weeks old.

The distribution of (pro)renin receptor/Atp6ap2 mRNA was examined in mouse kidney using hand-dissected nephron segments by semi-quantitative RT-qPCR. (P)RR/Atp6ap2 mRNA was detected in the glomerulus and all other segments, with highest levels in the connecting tubule/cortical collecting duct (CNT/CCD) and outer medullary collecting duct (OMCD) (Fig 1). The high mRNA abundance of (P)RR/Atp6ap2 in CNT/CCD and OMCD was paralleled by high mRNA levels of the B1 (Atp6v1b1) H^+^-ATPase subunit which is selectively enriched in intercalated cells [40,41]. The enrichment of nephron segments was ascertained by RT-qPCR for segment specific markers (Podoplanin for the glomerulum, NaPi-.IIa for the S1 /S2, S3 segments of the proximal tubule and DCT, NKCC2 for the TAL, NCC for the DCT, AQP2 for the collecting duct system, and pendrin for the CNT/CCD). Patterns of expression are in good agreement with previous transcript analyses along the mouse nephron [42].

**Fig 2:**
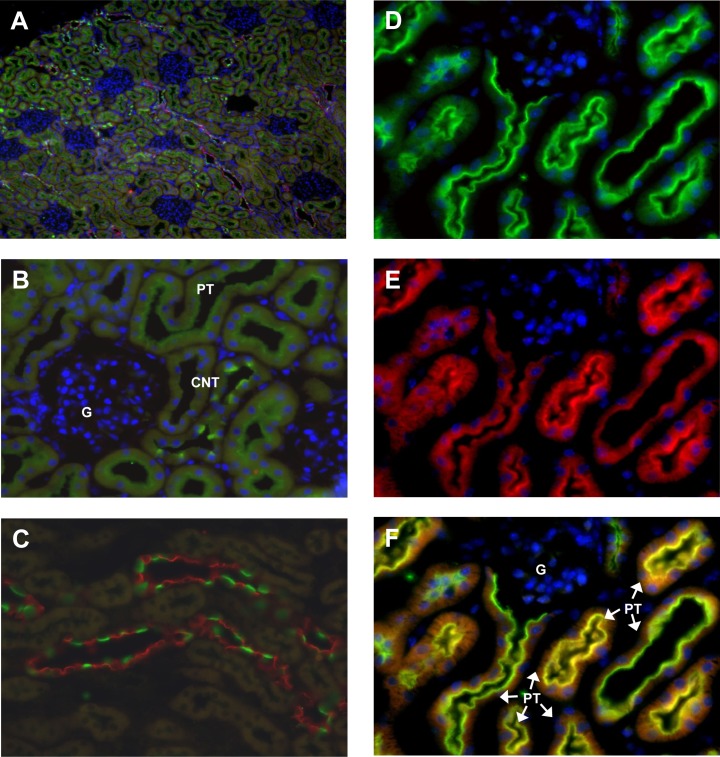
Localization of the (P)RR/Atp6ap2 in mouse kidney. Immunoflourescence staining for the (P)RR/Atp6p2 in mouse kidney (green). **(A)** Overview showing (P)RR/Atp6ap2 (green), the principal cell specific AQP2 water channel (red), and nuclei (blue), original magnification 40 x. **(B)** A cortical field with glomerulus (G), and (P)RR/Atp6ap2 staining (green) in the proximal tubule (PT) and connecting tubules (CNT), 400 x magnification. **(C)** Cortical and outer medullary collecting duct stained for (P)RR/Atp6ap2 (green) and the principal cell specific AQP2 water channel (red), 400 x magnification. **(E,D,F)** (P)RR staining (D: green) was detected in the proximal tubule (PT) in the brush border membrane and colocalized with the a4 H^+^-ATPase subunit (ATP6V0A4)(E: red) as indicated by the yellow color (F), original magnification 400x.

**Fig 3:**
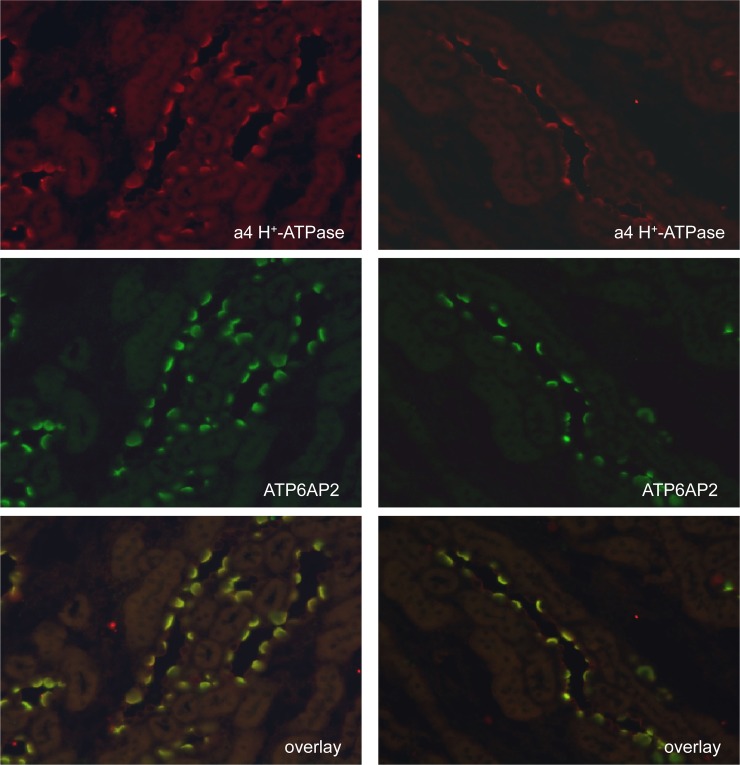
Colocalization of the (P)RR/Atp6ap2 with the a4 H^+^-ATPase subunit in intercalated cells. Staining of mouse kidney sections with antibodies against the a4 (Atp6v0a4) H^+^-ATPase subunit (red, upper panels) and the (P)RR/Atp6ap2 (green, middle panels) demonstrates colocalization (yellow, lower panels) in intercalated cells at the light microscopy level. Original magnification 400x.

Immunohistochemistry detected weak (P)RR/Atp6ap2 related staining in the glomerulus as described before [43,44]. However, clear signals were detected in the proximal tubule at the brush border membrane and in cells in the collecting duct system (Fig 2A and 2B). Costaining of (P)RR/Atp6ap2 with the a4 (Atp6v0a4) H^+^-ATPase subunit, which is expressed along the entire nephron [32,45,46], showed strong overlay at the apical side of proximal tubular cells (Fig 2D–2F). In the collecting duct, costaining with the principal cell specific marker AQP2 demonstrated that the (P)RR/Atp6ap2 was expressed in intercalated cells (Fig 2C). Further studies demonstrated that the (P)RR/Atp6ap2 colocalized in intercalated cells with the a4 (Atp6v0a4) H^+^-ATPase subunit that forms part of the plasma membrane H^+^-ATPase (Fig 3) [9,47].

**Fig 4:**
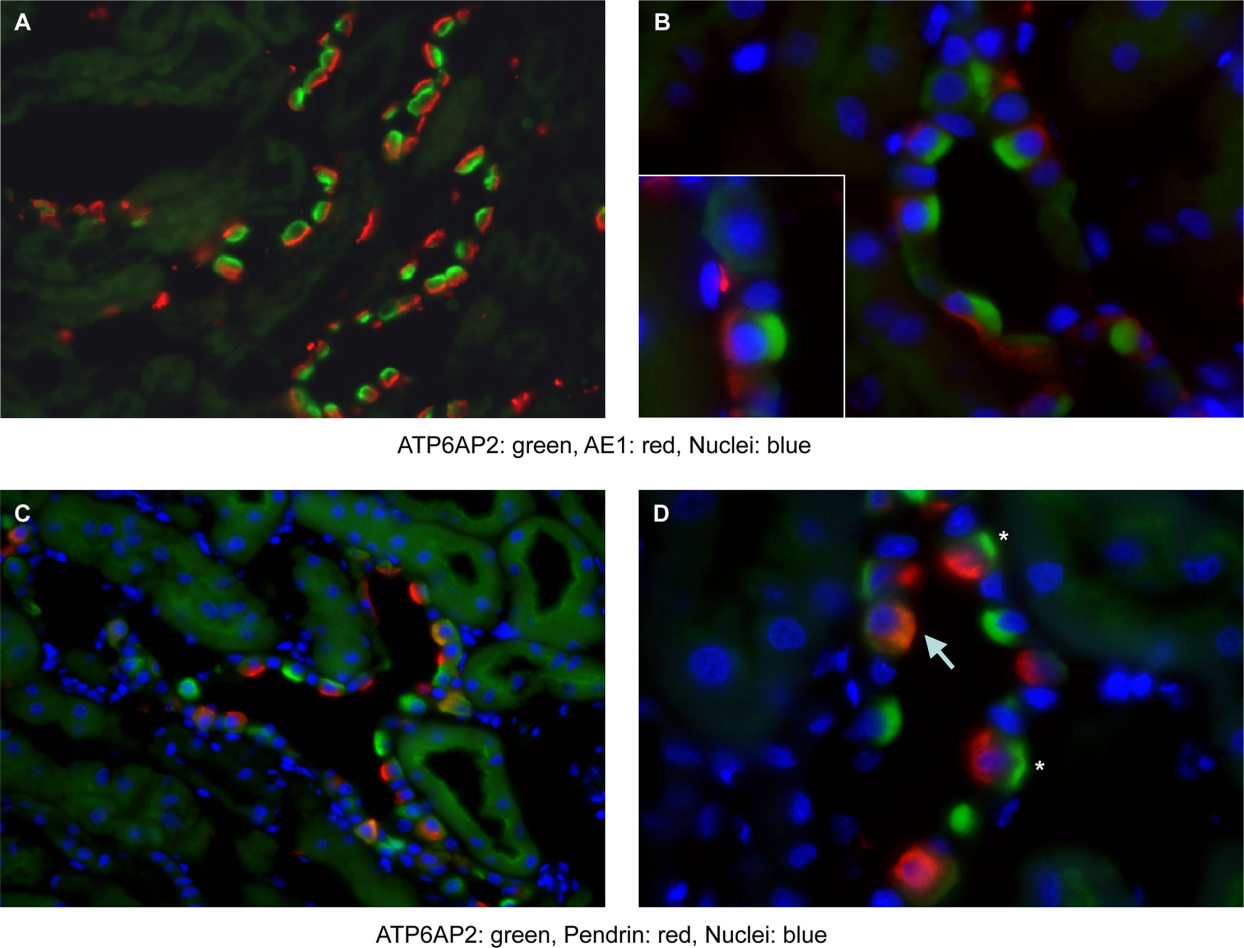
Subcellular localization of the (P)RR/Atp6ap2 in type A and non-type A intercalated cells. (A,B) Staining of mouse kidney sections with antibodies against the (P)RR/Atp6ap2 (green), the type A intercalated cell specific marker AE1 (red) and nuclei with DAPI (blue). In cells expressing AE1, (P)RR/Atp6ap2 related staining is found and localizes to the apical side of cells (insert in B), original magnification 630–1000 x. (C,D) Staining of mouse kidney sections with antibodies against the (P)RR/Atp6ap2 (green), the non-type A intercalated cell specific marker pendrin (red) and nuclei with DAPI (blue). In cells positive for pendrin, (P)RR/Atp6ap2 staining is detected either at the basolateral side of cells (asterisks in D) and/or luminal side (yellow overlay, arrow in D). Original magnification 400–630 x.

H^+^-ATPases in intercalated cells can be associated with the luminal membrane in type A intercalated cells and with either the basolateral and/or luminal membrane in non-type A intercalated cells [7,8,32,48,49]. Type A intercalated cells were identified by the presence of AE1 specifically expressed at the basolateral membrane of these cells. In AE1 positive cells, (P)RR/Atp6ap2 staining was always detected at the luminal side of cells (Fig 4A and 4B). In cells expressing pendrin, a marker of non-type A intercalated cells which is localized at the luminal membrane, (P)RR/Atp6ap2 staining was observed either at the basolateral membrane and/or colocalizing with pendrin at the luminal pole (Fig 4C and 4D).

Thus, the (P)RR colocalizes with H^+^-ATPases at the plasma membrane in various nephron segments and can be found both at the apical side of cells in the proximal tubule and in type A and non-type A intercalated cells as well as at the basolateral side of some non-type A intercalated cells as previously described for other H^+^-ATPase subunits [32,40,41,46,48,49].

### Regulation of (P)RR/Atp6ap2 mRNA and protein abundance in kidney by acid-base status and electrolyte intake

Mice were treated with different diets to alter activity of the different subtypes of intercalated cells and renal handling of bicarbonate and protons, conditions that have been associated with altered activity, expression, and/or localization of H^+^-ATPases along the nephron [32,48].

**Fig 5:**
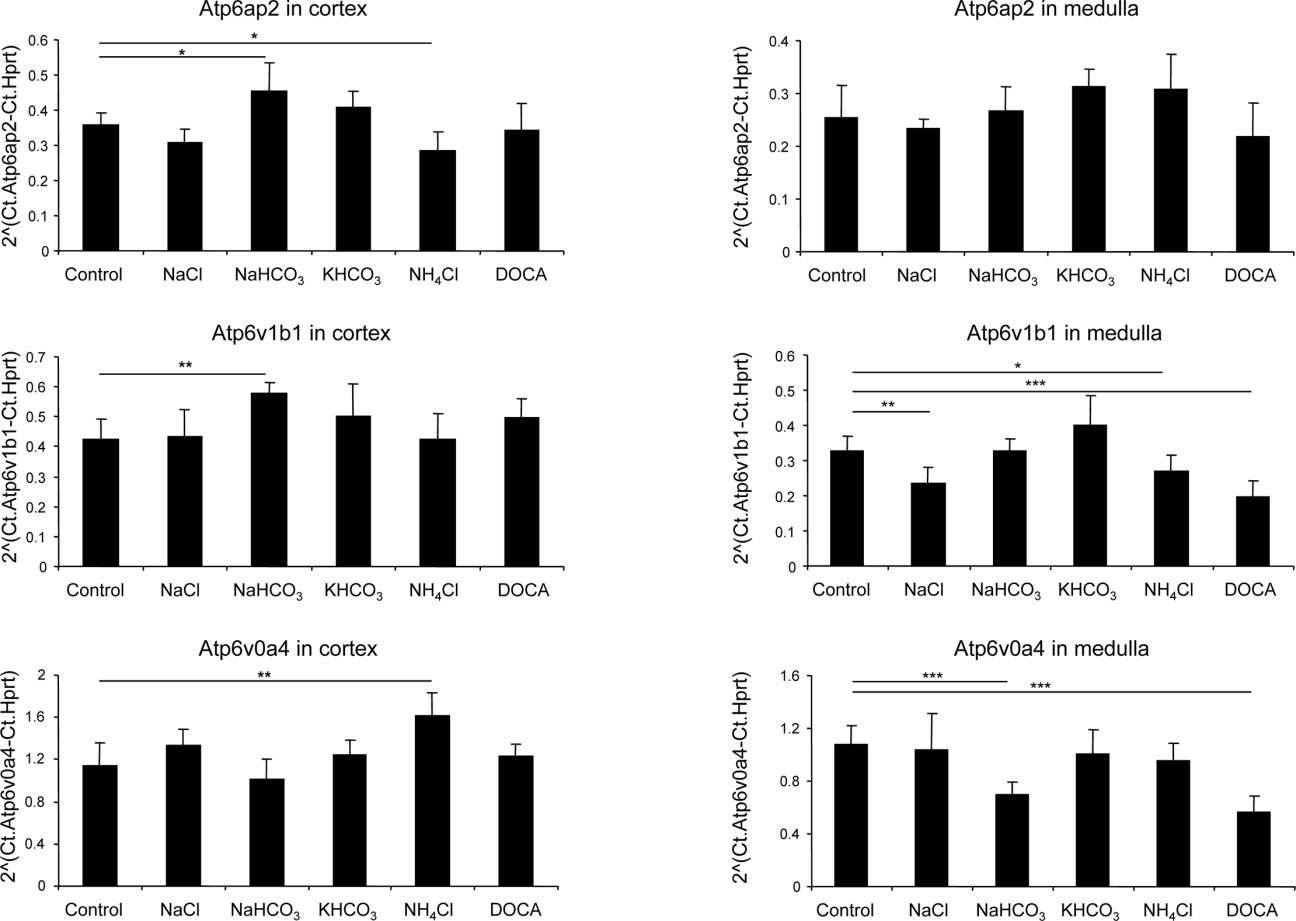
Regulated mRNA expression of (P)RR/Atp6ap2 and the B1/Atp6v1b1 and a4/Atp6v0a4 H^+^-ATPase subunits in mouse kidney. Mice were treated with NaCl, NaHCO_3,_ KHCO_3_, NH_4_Cl or DOCA for 7 days, and relative mRNA expression levels of (P)RR/Atp6ap2, and the B1/Atp6v1b1 and a4/Atp6v0a4 H^+^-ATPase subunits were assessed by semi-quantitative real-time RT-PCR in cortex and medulla. N = 5 animals/ group, *p ≤ 0.05, **p ≤ 0.01, ***p ≤ 0.001.

Semi-quantitative qPCR was used to assess the relative abundance of mRNAs encoding (P)RR, B1 and a4 subunits of the vacuolar H^+^-ATPase in dissected cortex and medulla from control mice and animals that had received NaCl, NaHCO_3_, KHCO_3_, NH_4_Cl, or the mineralocorticoid DOCA for 7 days (n = 5 per group). The abundance of (P)RR/Atp6ap2 mRNA was significantly increased in kidney cortex from NaHCO_3_ treated mice and reduced with NH_4_Cl treatment (Fig 5). Atp6v1b1 mRNA was higher in cortex with NaHCO_3_ treatment and Atp6v0a4 mRNA increased with NH_4_Cl supplementation (Fig 5). In kidney medulla, (P)RR/Atp6ap2 mRNA was not altered by any of the diets, whereas Atp6v1b1 mRNA decreased with NaCl, NH_4_Cl, and DOCA treatments. Similarly, Atp6v0a4 mRNA expression in medulla was reduced with NaHCO_3_ or DOCA treatments. Thus, these data provided no evidence for coordinated regulation of mRNA expression of these three genes.

**Fig 6:**
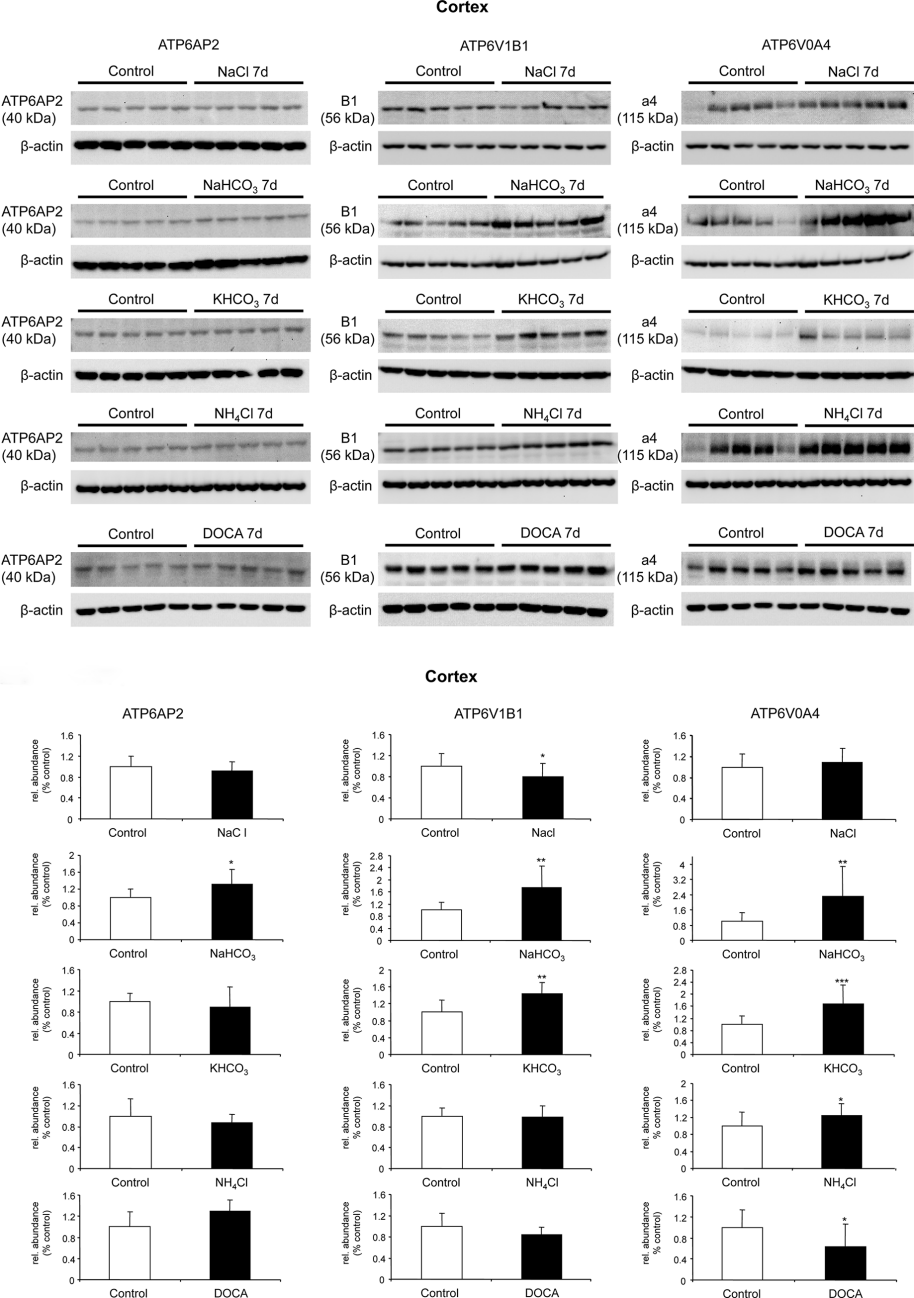
Regulated protein abundance of (P)RR/Atp6ap2 and the B1/Atp6v1b1 and a4/Atp6v0a4 H^+^-ATPase subunits in mouse kidney cortex. Mice were treated with NaCl, NaHCO_3,_ KHCO_3_, NH_4_Cl or DOCA for 7 days, and protein expression levels of (P)RR/Atp6ap2, and the B1/Atp6v1b1 and a4/Atp6v0a4 H^+^-ATPase subunits were examined by immunoblotting of total membrane fractions prepared from kidney cortex **(A)**. All blots were stripped and reprobed for β-actin. (42 kDa), and the ratio of (P)RR: β-actin was calculated. Bar graphs **(B)** summarize data from the blots. Arithmetic means ± SD are shown, n = 5 animals/ group, *p ≤ 0.05, **p ≤ 0.01, ***p ≤ 0.001.

**Fig 7:**
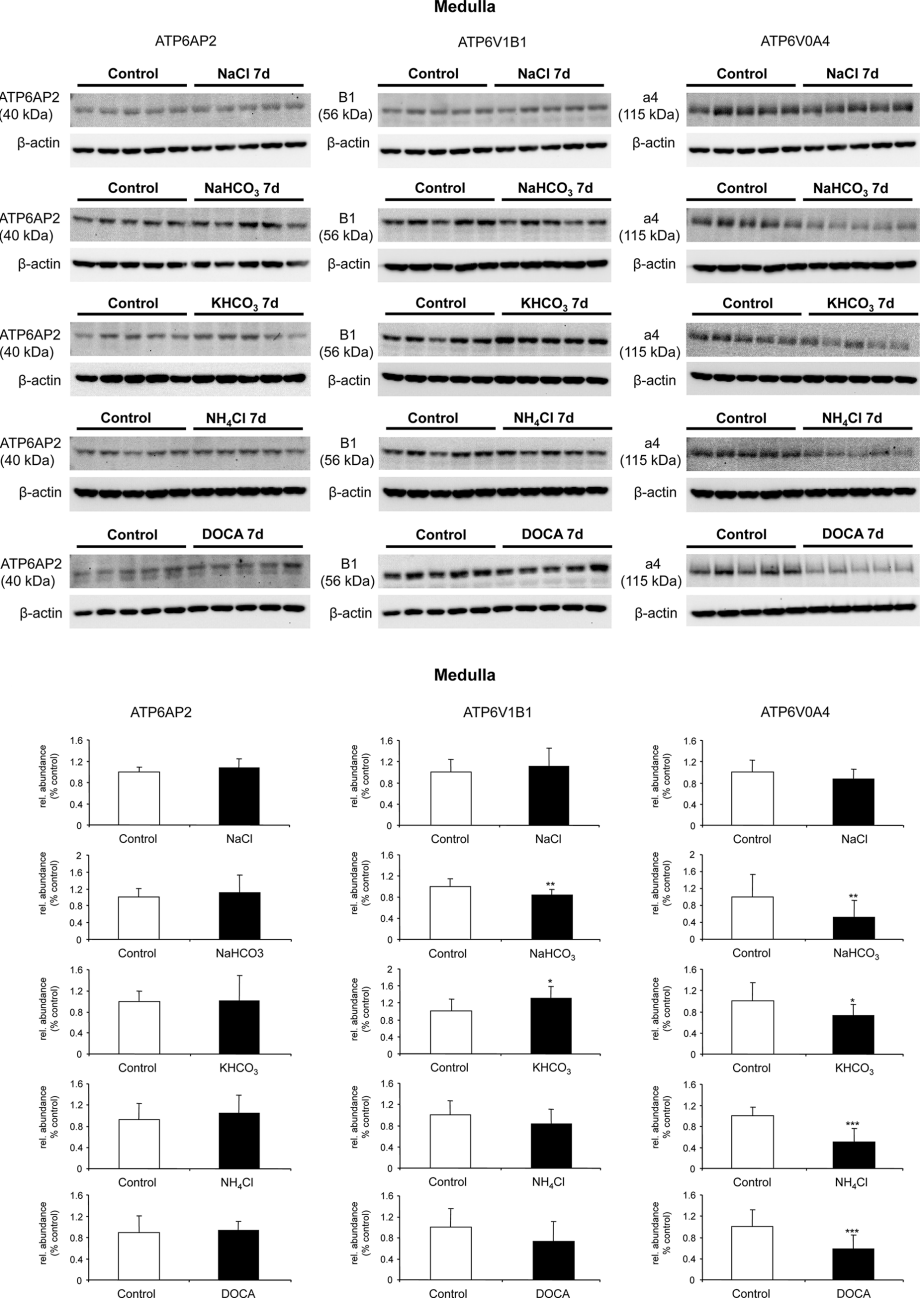
Regulated protein abundance of (P)RR/Atp6ap2 and the B1/Atp6v1b1 and a4/Atp6v0a4 H^+^-ATPase subunits in mouse kidney medulla. Mice were treated with NaCl, NaHCO_3,_ KHCO_3_, NH_4_Cl or DOCA for 7 days, and protein expression levels of (P)RR/Atp6ap2, and the B1/Atp6v1b1 and a4/Atp6v0a4 H^+^-ATPase subunits were examined by immunoblotting of total membrane fractions prepared from kidney outer and inner medulla **(A)**. All blots were stripped and reprobed for β-actin. (42 kDa), and the ratio of (P)RR to β-actin was calculated. Bar graphs **(B)** summarize data from the blots. Arithmetic means ± SD are shown, n = 5 animals/ group, *p ≤ 0.05, **p ≤ 0.01, ***p ≤ 0.001.

Next we assessed protein abundance of (P)RR/Atp6ap2, and the B1 and a4 H^+^-ATPase subunits separately in kidney cortex and medulla. In cortex, NaHCO_3_ treatment resulted in a significant increase in the protein abundance of (P)RR protein whereas all other treatments had no influence (Fig 6). The expression of the B1 (Atp6v1b1) subunit was reduced by NaCl intake and increased by alkali treatment such as NaHCO_3_ or KHCO_3_, whereas NaHCO_3,_ KHCO_3_, and NH_4_Cl increased the a4 (Atp6v0a4) subunit. In contrast, DOCA reduced a4 protein expression. In medulla, (P)RR/Atp6ap2 abundance was not regulated, whereas B1 abundance increased with KHCO_3_ and decreased with NaHCO_3_ treatment. Moreover, a4 expression decreased with NaHCO_3_, KHCO_3_, NH_4_Cl and DOCA treatments (Fig 7). Similar to the mRNA data, these experiments did not indicate coordinated regulation of protein abundance of H^+^-ATPase subunits and (P)RR/Atp6ap2.

### Trafficking of H^+^-ATPase and (P)RR/Atp6ap2 in response to alkali and acid loading

**Fig 8:**
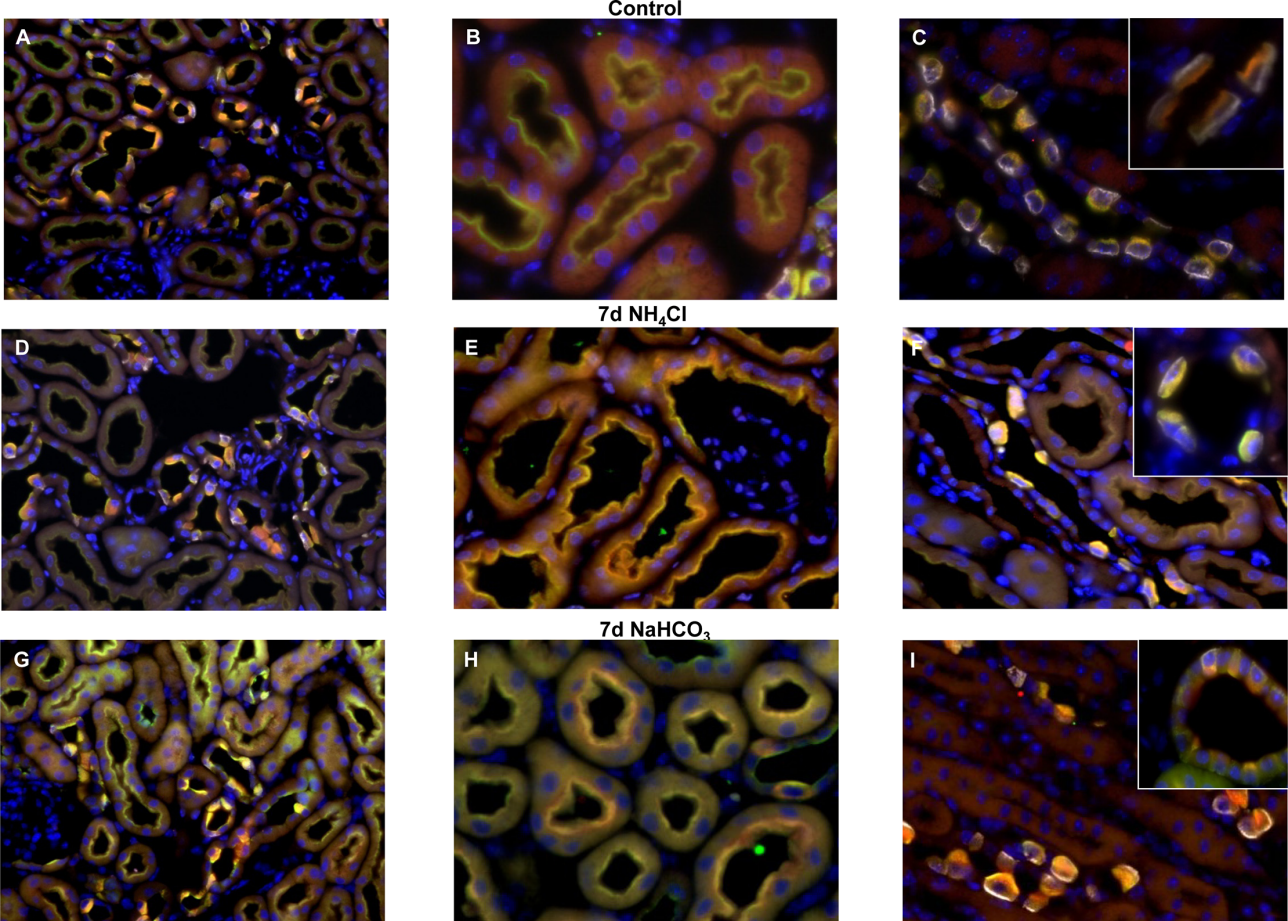
Colocalization of (P)RR/Atp6ap2 and the a4/Atp6v0a4 H^+^-ATPase subunit under conditions of acidosis and alkalosis. Mice were left untreated or received NH_4_Cl (0.28 M) or NaHCO_3_ (0.28 M) in drinking water for 7 days to induce metabolic acidosis or alkalosis, respectively. Kidney sections were stained with antibodies against the (P)RR/Atp6ap2 (green), the a4/Atp6v0a4 H^+^-ATPase subunit (red), the type A intercalated cell specific anion exchanger AE1 (white), and with DAPI (blue) to mark nuclei. **(A-C)** Kidney sections from control mice with a cortical field (A) (400x), convoluted proximal tubules (B)(630 x), and an outer medullary collecting duct (C)(630x). The insert in (C) shows a higher magnification of type A intercalated cells in the outer medulla. **(D-F)** Kidney sections from mice receiving NH_4_Cl showing a cortical field (D) (400x), convoluted proximal tubules (E)(630 x), and an outer medullary collecting duct (F)(630x). The insert in (F) shows a higher magnification of type A intercalated cells in the outer medulla. **(G-I)** Kidney sections from mice treated with NaHCO_3_ showing a cortical field (G) (400x), convoluted proximal tubules (H)(630 x), and an outer medullary collecting duct (I)(630x). The insert in (I) shows a higher magnification of type A intercalated cells in the outer medulla. N = 4 animals/group.

Loading of rodents with either alkali/bicarbonate or acid (NH_4_Cl) provokes increased trafficking of H^+^-ATPase subunits to the basolateral membrane of non-type A intercalated cells or luminal membrane of type A intercalated cells, respectively [32,48,49]. We performed immunohistochemistry for the a4 H^+^-ATPase subunit together with the (P)RR/ Atp6ap2 and AE1 as a marker of type A intercalated cells on kidneys from control mice and mice receiving NaHCO_3_ or NH_4_Cl for 7 days (Fig 8). In the proximal tubule, the signals for (P)RR/Atp6ap2 (green) and a4 H^+^-ATPase (red) showed a high degree of colocalization as indicated by the strong yellow color (Fig 8B, 8E and 8H). Similarly, in intercalated cells, (P)RR and a4 H^+^-ATPase strongly colocalized at the luminal and/or basolateral side (Fig 8A, 8C, 8D, 8F, 8G and 8I). In AE1 positive intercalated cells, yellow staining was confined mostly to the luminal membrane, whereas in intercalated cells negative for AE1, yellow staining was detected at the luminal and/or basolateral side. Neither NaHCO_3_ nor NH_4_Cl treatment did alter the apparent costaining, at least at the level of light microscopy.

### Acute exposure of *ex vivo* microperfused collecting ducts to prorenin does not stimulate H^+^-ATPase activity and ERK1/2 phosphorylation

**Fig 9:**
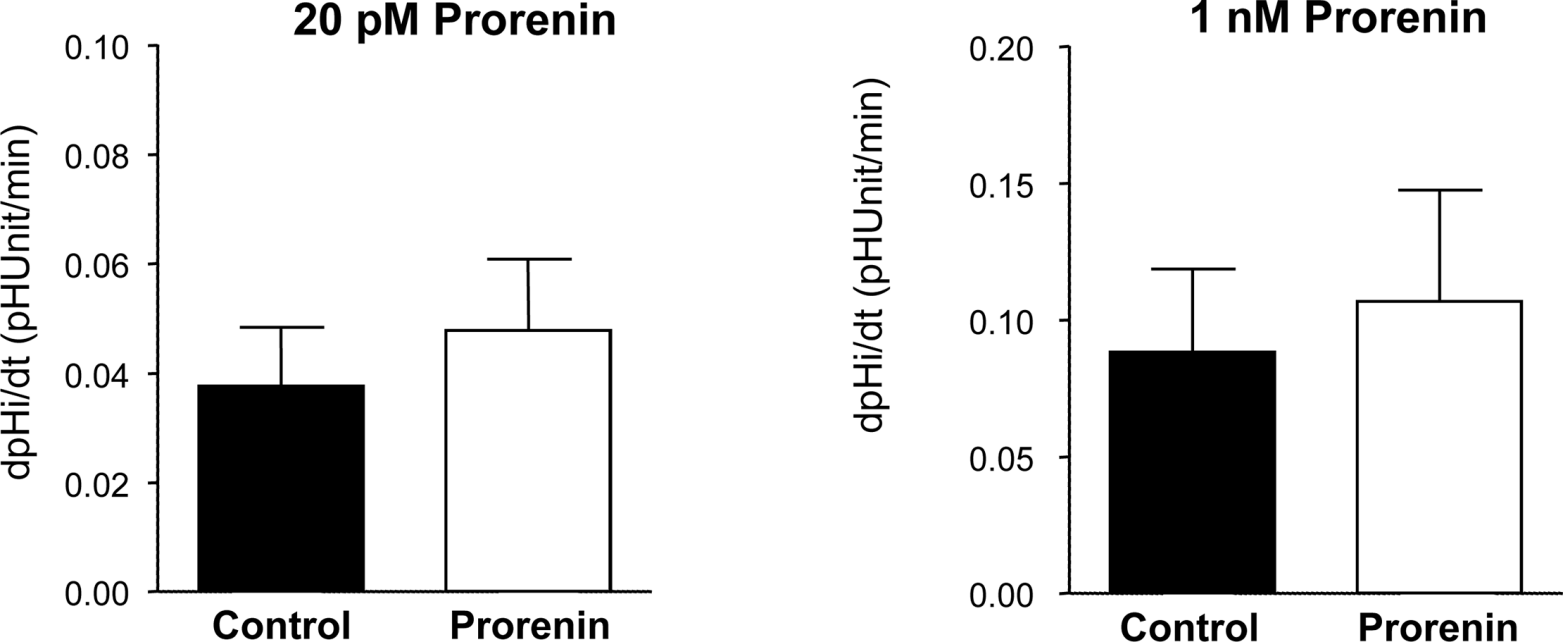
Prorenin does not acutely stimulate H^+^-ATPase activity in microperfused mouse outer medullary collecting ducts. Cortical collecting ducts were prepared by hand-dissection from mouse kidney and microperfused *ex vivo*. H^+^-ATPase activity was assessed from pH_i_ recovery rates in BCECF loaded intercalated cells using the NH_4_Cl prepulse technique. 1 nM or 20 pM Prorenin was applied from the luminal side and pH_i_ recovery rates were measured. N = 4 to 6 tubules/group.

**Fig 10:**
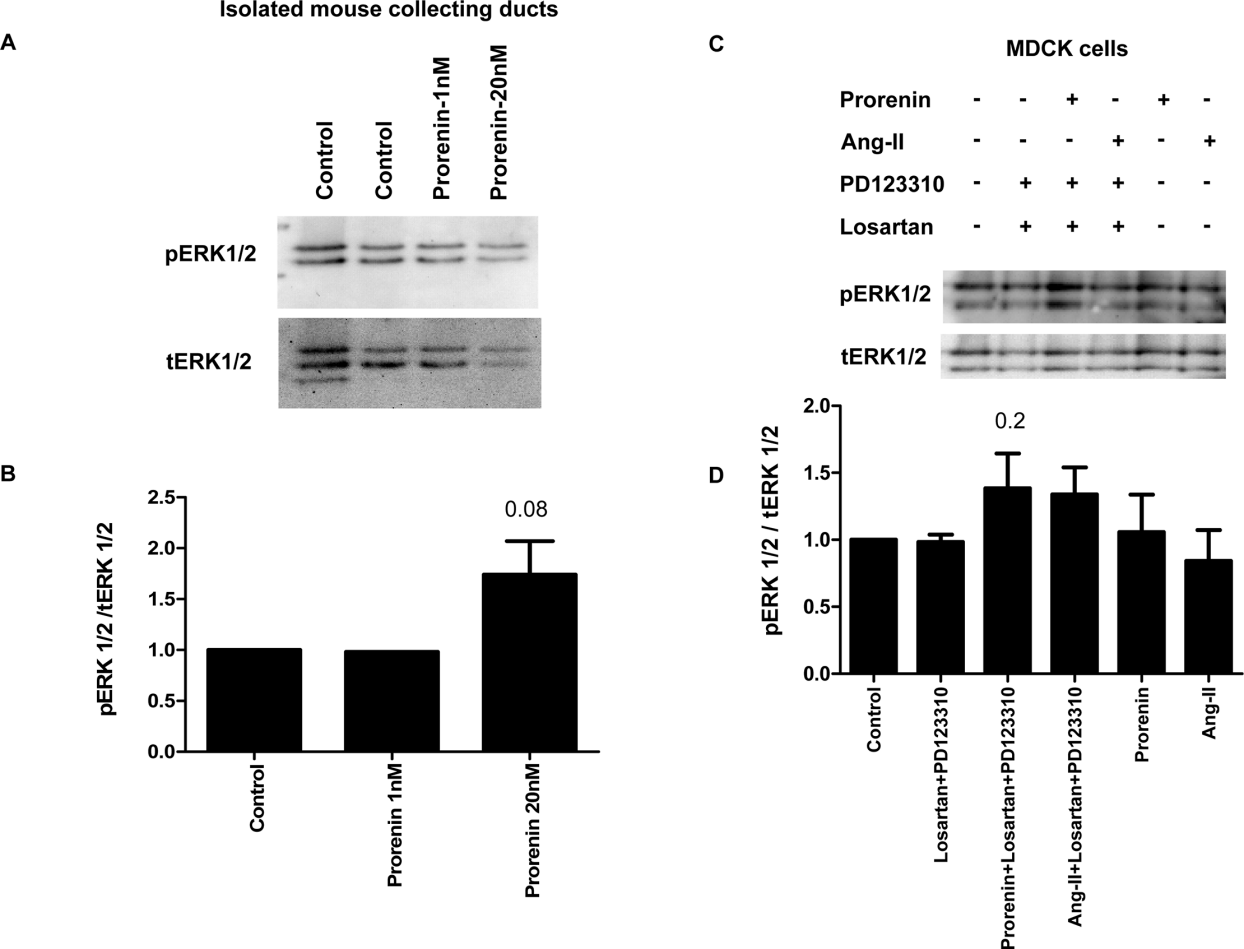
Prorenin stimulates ERK1/2 phosphorylation in isolated mouse collecting ducts and MDCK cells. **(A)** Outer medullary collecting ducts were prepared by hand-dissection from kidneys of mice expressing EGFP under the control of the intercalated cell specific Atp6v1b1 promoter [26] and incubated *in vitro* with or without prorenin for 10 min. Immunoblots were performed for pERK1/2 and total ERK1/2. **(B)** Three independent experiments were performed and are summarized as bar graph showing the ratio of pERK1/2 over total ERK1/2. **(C, D)** MDCK cells were incubated for 10 minutes with prorenin (20 nM) or angiotensin II (10 nM) in the absence or presence of the AT_1_ and AT_2_ receptor blockers losartan (10 μM) and PD123310 (10 μM). Immunoblots were performed for pERK1/2 and total ERK1/2. Three independent experiments were performed and are summarized as bar graph showing the ratio of pERK1/2 over total ERK1/2.

The (P)RR/Atp6ap2 has been identified because of its ability to bind prorenin and experiments in MDCK cells demonstrated increased ERK1/2 phosphorylation and stimulated H^+^-ATPase activity when incubated with prorenin [50,51]. Thus, we tested in freshly isolated and microperfused mouse cortical collecting ducts whether luminal application of prorenin could stimulate H^+^-ATPase activity also in *ex vivo* preparations (Fig 9). However, luminal microperfusion with two different concentrations of prorenin (20 pM and 1 nM) for 15 minutes did not alter H^+^-ATPase activity measured as pH_i_ recovery rates after intracellular acidification with a NH_4_Cl prepulse (20 mM) as described previously [13,20,21,29]. Next, we incubated hand-dissected connecting tubules and cortical collecting ducts with prorenin (1 and 20 nM) for 10 min at 37°C *in vitro*. For these experiments, we used mice expressing eGFP in intercalated cells [26] to facilitate isolation of large enough quantities of these nephron segments for experiments. In three independent preparations, no difference in pERK1/2 abundance could be detected (Fig 10A and 10B), however, 20 nM prorenin caused a strong trend towards increased ERK1/2 phosphorylation (p = 0.08). We performed parallel experiments in MDCK cells incubated for 10 minutes with prorenin (20 nM) or angiotensin II (10 nM) in the absence or presence of the AT_1_ and AT_2_ receptor blockers losartan (10 μM) and PD123310 (10 μM) (Fig 10). In the combined presence of losartan and PD123310 a non-significant tendency to more pERK1/2 abundance (p = 0.2) was detected in MDCK cells incubated with prorenin (Fig 10C and 10D).

## Discussion

Our study provides new insights into the localization of the prorenin receptor/Atp6ap2 and its regulation in mouse kidney by confirming and expanding previous observations reporting the expression and localization of the (P)RR/Atp6ap2 in the vasculature and podocytes of the glomerulus [4,43,44,52], in proximal tubule [52,53], and in the collecting duct [52] and intercalated cells [50,54,55,56].

Here, we present the detailed expression and localization of the (P)RR along the entire nephron. Semi-quantitative qPCR showed abundant expression of (P)RR/Atp6ap2 mRNA in hand-dissected mouse nephron segments spreading from glomerulus to the distal segments including the collecting duct. Of note, the highest mRNA levels were found in the collecting duct system coinciding with the localization of intercalated cells. Previous experiments using in-situ hybridization in rat kidney had detected signals in proximal tubule and thick ascending limb of the loop of Henle (TAL) but with much weaker intensity than in the collecting duct supporting our observations [50]. Immunohistochemistry shows (P)RR/Atp6ap2 localization at the luminal membrane of proximal tubules and in all subtypes of intercalated cells. Weak immunohistochemical signals for (P)RR/Atp6ap2 were also detected in TAL and distal convoluted tubule (DCT) and were very weak or absent from segment-specific cells (principal cells) in the collecting duct system. In intercalated cells, localization of the (P)RR/Atp6ap2 differed between specific cell subtypes. In type A intercalated cells, identified by positive basolateral localization of AE1 [57], (P)RR/Atp6ap2 staining was detected only at the luminal pole consistent with the luminal localization of H^+^-ATPases in these cells [7,49]. Consistently, Advani and colleagues had reported the localization of immunogold-labeled (P)RR/Atp6ap2 at the luminal membrane of rat type A intercalated cells with electron microscopy [50]. In contrast, intercalated cells expressing pendrin (type B and non-A/non-B intercalated cells) [58,59] showed luminal, basolateral or combined (P)RR/Atp6ap2 staining as reported previously also for other H^+^-ATPase subunits [32,46,48,49]. Accordingly, (P)RR/Atp6ap2 staining colocalized at the level of light microscopy with the a4/Atp6v0a4 H^+^-ATPase subunit at the brush border membranes of proximal tubule cells and in all subtypes of intercalated cells. Thus, the (P)RR/Atp6ap2 is apparently localized to kidney cells where plasma membrane-associated H^+^-ATPases are also expressed such as in the proximal tubule and intercalated cells.

Next we examined whether mRNA and protein abundance of the (P)RR/Atp6ap2 paralleled mRNA and protein expression levels of two H^+^-ATPase subunits, a4/Atp6v0a4 and B1/Atp6v1b1, in membrane preparations from kidney cortex and kidney medulla from mice subjected to different treatments known to affect acid-base status and H^+^-ATPase regulation [25,32,35,48,60,61]. Changes in acid-base and electrolyte balance caused changes in mRNA and protein expression of all three molecules but did not show an uniform and obvious pattern of coregulation. At the level of the kidney cortex this may not be surprising since the preparation contains a mixture of proximal tubules, connecting tubules, and cortical collecting ducts which may have differential patterns of regulation. The medullary preparations (from outer and inner medulla) are more homogenous and thus representative for medullary collecting ducts even though containing also fractions from the late proximal tubule and medullary TALs [62]. Nevertheless, no consistent coregulation of the H^+^-ATPase subunits and the (P)RR/atp6ap2 was observed. Along the same line, B1 and a4 subunit isoforms showed varying responses which may reflect different expression patterns. Part of the changes in H^+^-ATPase subunit isoform expression and (PRR/Atp6ap2 may also be due to remodeling of the collecting duct that during chronic changes in electrolyte and acid-base status can affect the relative abundance of principal versus intercalated cells as well as the relative frequency of the different intercalated cell subtypes [25,63].

Regulation of the (P)RR/Atp6ap2 has been shown in rodent models subjected to low or high salt intake [52,64]. Similar to our results no effect of high salt intake on (P)RR/Atp6ap2 mRNA was detected [52] whereas low salt diet increased (P)RR/Atp6ap2 mRNA [64,65]. In contrast, on protein level high salt diet was associated with increased full length (P)RR/Atp6ap2 protein expression in cortex and medulla which was attributed to changes in glomerular and proximal tubular abundance [52]. But also low salt intake caused higher expression of (P)RR/Atp6ap2 protein at the level of the total kidney [64,65,66]. The functional relevance of the seemingly same response of the (P)RR/Atp6ap2 to both high and low salt intake are not known to date.

Regulation of H^+^-ATPase activity occurs on several levels involving assembly and disassembly of V_0_ and V_1_ sectors, trafficking of pumps into and from the membrane, and phosphorylation of subunits [6,7]. Changes in acid-base status or electrolyte homeostasis as well as aldosterone or its analogue DOCA have been shown to induce marked redistribution of H^+^-ATPases in intercalated cells with more pronounced membrane association at the luminal membrane in type A intercalated cells upon NH_4_Cl or DOCA treatment [13,20,32,48,67]. Likewise, supplementation with bicarbonate leads to a strong staining of basolateral and/or luminal membranes in type B and non-A/non-B intercalated cells [32,48,67]. Thus, we tested whether the (P)RR/Atp6ap2 would colocalize with the a4/Atp6v0a4 H^+^-ATPase subunit that has been previously shown to participate in the trafficking of H^+^-ATPases to the different membrane domains in type A and non-type A intercalated cells [32]. Detailed analysis of intercalated cells in the connecting tubule and medullary collecting duct showed that (P)RR/Atp6ap2 and the a4/Atp6v0a4 H^+^-ATPase subunit showed a high degree of colocalization both in intercalated cells stained for AE1 (type A intercalated cells) and intercalated cells negative for AE1 (non-type A intercalated cells). In the latter cell population, (P)RR/Atp6ap2 and a4/Atp6v0a4 were detected at the basolateral and luminal membrane as expected and consistent with the detection of (P)RR/Atp6ap2 in pendrin positive cells (Fig 4C and 4D). Also in the proximal tubule, (P)RR/Atp6ap2 and a4/Atp6v0a4 strongly colocalized at the brush border membrane under all treatments. Thus, the (P)RR/Atp6ap2 appears to colocalize with H^+^-ATPases under different conditions inducing a subcellular redistribution of pumps.

In a last set of experiments we addressed the question whether prorenin would acutely affect basal H^+^-ATPase function in type A intercalated cells. Isolated cortical collecting ducts microperfused *in vitro* were exposed to two different concentrations of luminal prorenin that had previously been shown to induce cellular responses in other preparations [50,51]. However, we detected no differences between tubules microperfused with prorenin or left untreated in the realkalinization rates after washing out the NH_4_Cl prepulse. The rate of realkalinization represents mostly proton extrusion by H^+^-ATPases as indicated by its sensitivity to typical H^+^-ATPase inhibitors such as bafilomycin or concanamycin [20,29,68]. Moreover, we and others have previously shown that various stimuli including aldosterone, angiotensin II, or cAMP can stimulate H^+^-ATPase activity in such preparations [12,13,20,21,69]. In a previous study using MDCK cells as model for intercalated cells we had shown that prorenin was able to stimulate H^+^-ATPase activity, albeit at high concentrations, and that siRNA mediated suppression of (P)RR/Atp6ap2 expression reduced expression of the a2 but not the a4, d2, and B1/2 H^+^-ATPase subunits and diminished stimulation of H^+^-ATPase activity by the antidiuretic hormone [51]. The discrepancy between our present study and the previous experiments in MDCK cells may be explained by differences in the composition of proton pump subunits present in MDCK and the native murine cortical collecting duct or by more general differences between the MDCK cell culture system and freshly isolated intercalated cells. Some subclones of the original MDCK cell line, namely the C7 and C11 clones, are believed to resemble principal and intercalated cells [28]. However, we performed qPCR for typical markers expressed by intercalated cells (e.g. pendrin, AE1, Foxi1, CP2L1, B1/ATP6V1B1) and by principal cells (e.g. AQP2) on these clones and found only very low or inconsistent expression of specific intercalated cell markers in these clones (Kampik, Wagner, unpublished data) suggesting that MDCK cells may not represent a very faithful model to investigate intercalated cell functions and regulation. Along the same line, Advani et al had reported that incubation of MDCK cells with renin and prorenin stimulated ERK1/2 phosphorylation and that this effect was blocked by the H^+^-ATPase inhibitor bafilomycin [50]. We thus incubated freshly isolated cortical collecting ducts and MDCK cells with prorenin. In both preparations, cortical collecting ducts and MDCK cells, only a weak but not significant increase of ERK1/2 phosphorylation was found. In freshly isolated murine collecting ducts ERK1/2 participates in the stimulatory effect of aldosterone on H^+^-ATPase activity [13]. Hence, an increase in pERK1/2 might directly stimulate H^+^-ATPase activity or be permissive for other positive stimuli. Therefore, the absence of a stimulatory effect of prorenin on H^+^-ATPase activity is consistent with the absence of an effect on ERK1/2 phosphorylation in the same preparation and possibly also in MDCK cells. Of note, all batches of prorenin were extensively tested for *in vitro* activity before use in microperfusion or *in vitro* incubation experiments ruling out that an inactive form of prorenin was used [70,71].

In summary, we extend previous observations on the localization of the (P)RR/Atp6ap2 along the entire murine nephron and provide detailed information on its subcellular localization in the various types of intercalated cells. (P)RR/Atp6ap2 and two other H^+^-ATPase subunits did not show coordinated regulation of mRNA and protein expression in kidneys from mice receiving different treatments. Nevertheless, immunolocalization of (P)RR/Atp6ap2 and the a4/Atp6v0a4 H^+^-ATPase subunit showed colocalization under all conditions suggesting that (P)RR/Atp6ap2 may be closely linked to or an integral part of the pump. In freshly isolated collecting ducts, prorenin had no effect on basal H^+^-ATPase activity and ERK1/2 phosphorylation indicating that prorenin and possibly also renin are not directly regulating H^+^-ATPase activity under the conditions used in this assay. Whether prorenin may have a permissive effect on the regulation of H^+^-ATPase function by other stimuli cannot be ruled out. Our data do not provide any answer to the question whether the (P)RR/Atp6ap2 is a functionally relevant part of the H^+^-ATPase in the proximal tubule or intercalated cells. Experiments in *Drosophila* suggest that the (P)RR/Atp6ap2 might participate in endocytic retrieval of proteins from urine in the proximal tubule [22]. Genetic deletion of (P)RR/Atp6ap2 in other tissues and cells suggests that the absence of (P)RR/Atp6ap2 impairs the expression and function of the proton pump complex [44,51,72]. Also, the genetic deletion of (P)RR/Atp6ap2 from the entire collecting duct in mice causes hydronephrosis and more alkaline urine consistent with an important role of the protein in the collecting duct [73]. The timed and specific deletion of (P)RR/Atp6ap2 from renal cells will have to address the specific function(s) of the (P)RR/Atp6ap2 in these cells and its relationship to H^+^-ATPase function.

## Supporting Information

S1 Table Sequences of forward and reverse primers and probes used for semi-quantitative real-time RT-PCR. (DOCX)
