# Supplementary material for: Colocalization of the (Pro)renin Receptor/Atp6ap2 with H+-ATPases in Mouse Kidney but Prorenin Does Not Acutely Regulate Intercalated Cell H+-ATPase Activity
Source: PLoS One. 2016 Jan 29;11(1):e0147831. doi: 10.1371/journal.pone.0147831 (PMC4732657; doi:10.1371/journal.pone.0147831)
Supplement: S1 Table — (DOCX) [file pone.0147831.s001.docx]

**S1 Table: Primer and probe sequences**

Forward and reverse primers and probes used for semi-quantitative real-time RT-PCR

|  | **Primer** | **Fw** | **Probe** |
| --- | --- | --- | --- |
|  |  | **Rv** |  |
| (P)RR | 5' ACG CAG TGG TAG AGT TAG TGA CTG 3' | | 5' TCC TTG AGG CAA AAC AAG AGA ACA CCC 3' |
|  | 5' AGT TGA AAA CCA CTG AAT ACT CCA A 3' | |  |
| ATP6V1B1 | 5' AGG ACA GTG TGC AGC GTC AAT 3' | | 5' CCC AGT ATG CTG AGA TTG TCA ACT TTA CCC TCC 3' |
|  | 5' CCT GAA CAA TGG CCT TGG TC 3' | |  |
| ATP6V0A4 | '5' AGC CAA GCA CCA GAA ATC TCA 3' | | '5' CTG CAG TCT TTC ACG ATC CAC GAG GA 3' |
|  | '5' GAG TGG TCA CCC TCC ACA GC 3' | |  |
| NaPi2a | 5' TGA TCA CCA GCA TTG CCG 3' | | '5' CCA GAC ACA ACA GAG GCT TCC ACT TCT ATG TC 3' |
|  | 5' GTG TTT GCA AGG CTG CCG 3' | |  |
| Podoplanin  (Pdpn) | Mm00494716_m1 (Applied Biosystem) | |  |
| NCC | 5' GGC TTT GCA GAA ACC GTA AGG 3' | | '5' TAC TGC AGG AGT ATG GCA CAC CCA TCG TA 3' |
|  | 5' ACA CCG ATG ATG CGG ATG T 3' | |  |
| NKCC2 | 5' TCA CCA CCG TGG CCT ACA TA 3' | | '5' CTA TTT GCG TAG CCG CCT GTG TGG TC 3' |
|  | 5' TTC ATG CTG CCA GTG GCA 3' | |  |
| Pendrin | 5' GCC TTT GGG ATA AGC AAC GTC 3' | | 5' TGG ATT TTT CTC CTG TTT TGT GGC TAC CAC T 3' |
|  | 5' CAA CGA TGG CAA CCA TCA CA 3' | |  |
| AQP2 | 5' TGG TGC TGT GCA TCT TTG CCT 3' | | 5' ACC TCC TTG GGA TCT ATT TCA CCG G 3' |
|  | 5' ACT TGC CAG TGA CAA CTG CTG 3' | |  |
| HPRT | 5' TTA TCA GAC TGA AGA GCT ACT GTA ATG ATC 3' | | 5' TGA GAG ATC ATC TCC ACC AAT AAC TTT TAT GTC CC 3' |
|  | 5' TTA CCA GTG TCA ATT ATA TCT TCA ACA ATC 3' | |  |
|  |  | |  |
